# Evidence of Malodorous Chloroanisoles in “Mold Houses” Was Omitted When Indoor Air Research Evolved

**DOI:** 10.3390/microorganisms13061363

**Published:** 2025-06-12

**Authors:** Johnny C. Lorentzen, Gunnar Johanson

**Affiliations:** Integrative Toxicology, Institute of Environmental Medicine, Karolinska Institutet, SE-171 77 Stockholm, Sweden; gunnar.johanson@ki.se

**Keywords:** asthma, allergy, sick building syndrome, pesticides, wood preservatives, confounding, odor, mold, dampness, indoor air

## Abstract

Herein, we address the peculiar lack of scientific reporting on odor potent chloroanisoles (CAs) in the built environment. We have searched and critically examined sources beyond peer-reviewed scientific journals, namely research conferences, parliamentary records, newspaper articles, and cartoons. We provide evidence that CAs evolved on a large scale in Swedish buildings in the early 1970s and evoked a typical sticky malodor that was attributed to mold and gave rise to the term “mold houses”. The term first appeared in Swedish newspapers in 1978, and the media attention increased rapidly. The malodorous “mold houses” reached the Swedish parliament and led to economic compensation for afflicted homeowners. The “mold houses” became “sick houses” as researchers, predominantly from Sweden, introduced and became world leaders on the “sick buildings syndrome” (SBS). Researchers became aware of the CAs but did not mention them in peer-reviewed articles, just as they did not mention a well-known source of the sticky malodor, namely, legacy preserved wood where CAs were formed through microbial methylation of toxic chlorophenols (CPs). Thus, the mold story from the early 1970s was maintained and prevented the malodorous CAs from becoming recognized as indicators of the presence of hazardous CPs. Our study is the first to report the impact of an indoor malodor, not only on a few people, but on society.

## 1. Introduction

After World War II (WWII) the broadly biocidal and cheap-to-produce chlorophenols (CPs), including pentachlorophenol (PCP), found many large-scale uses. The high toxicity of CPs for, e.g., humans, farm animals and wildlife, and, in particular, of PCP, was recognized early [1,2,3,4,5,6], see e.g., reviews by the World Health Organization (WHO) in the 1980s [3,4]. Acute effects in experimental animals include mucosal irritation (eyes, nose, and throat), skin damage (swelling, hair loss, and flushed skin areas), and uncoupling of oxidative phosphorylation, resulting in fever. Repeated exposure may result in reduced growth rate and serum thyroid level; increased liver weight and liver enzyme activities; histological effects in liver, spleen, and kidney; hematological and immunological effects; fetotoxicity; and impaired renal function [3,4]. Today, PCP is classified as “Carcinogenic to humans” (Group 1) and 2,4,6-trichlorophenol as “Possibly carcinogenic to humans” (Group 2B) by the International Agency for Research on Cancer (IARC) [7]. PCP and its salts and esters are listed as persistent organic pollutants (POPs), Annex A (Elimination) by the Stockholm Convention [8].

**Figure 1:**
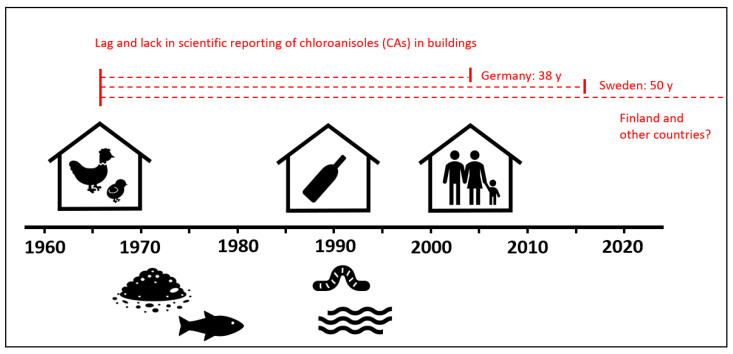
Timeline of the first scientific reports of chloroanisoles (CAs) in various environments [9,10,11,21,25,29,30,31,32,33,34]. A comparison is made (in red) between the first report concerning houses for chicken in 1966 [21], and humans in 2004 [34] in Germany, Sweden in 2016 [25], and other countries where CAs remain unreported in the scientific domain, such as Finland.

The CPs soon emerged as widespread contaminants in various natural environments and it became apparent that some microorganisms can methylate the CPs into chloroanisoles (CAs), when CPs occur in soil [9], water [10], and wildlife, for example, fish [11]. The formed CAs evoke a typical sticky malodor at very low concentrations [12] and became notorious for spoiling the smell and taste of various produce, such as foods [13], wine and other beverages [14,15,16,17], tobacco [18], and pharmaceuticals [19]. Often, the consumer items were tainted during indoor transportation of goods on CP-treated wood pallets and freight container floors [20]. Concerning indoors tainting, CAs were also highly problematic in buildings for chicken [21,22,23,24] and wine, causing sensory defects in the products. Unfortunately, CAs also evolved in buildings for humans, such as kindergartens, schools, offices, and homes, due to building practices applied during the post-WWII building booms. In the late 1970s, countries started to ban the CPs, with Sweden being first. Before that, industry and government agencies, for several decades, promoted the use of CPs in damp conditions to prevent mold on interior surfaces and wood decay fungi in house constructions. This created the perfect conditions for large scale odor formation as microbial growth was not always completely blocked, and microscopic amounts of mold were sufficient to produce and emit enough CAs to evoke the malodor [12,25,26,27]. In Sweden, nationwide malodor evolved in the early 1970s but even though measurements of CAs were marketed in 1999 [28], the malodorous microbial molecules were not reported in any peer-reviewed journal until 2016 [25]. In Finland, the CAs still have not been scientifically reported even though their presence indoors is now acknowledged by the Finnish National Public Health Institute. This is remarkable, considering that Finnish and Swedish researchers reported CAs in worms [29,30] and fish [31] more than 30 years ago. Evidently, there is a peculiar lag and even lack of scientific reporting on CAs in the built environment for humans (Figure 1).

In Sweden, the lag of scientific reporting is likely due to the fact that the presence of CPs in Swedish buildings was not officially recognized. When the CPs were banned in 1977, governmental agencies communicated the decision as being based on concern for the environment and workers’ health [12]. Sweden had world-leading toxicological expertise on CPs at the time, yet a Swedish review from 1980 did not mention CPs in buildings and the resulting indoor exposure [35]. This silence in the scientific domain was maintained until our first articles on the subject were published in 2016 [12,25,26,27].

With the presence of CPs in buildings not officially recognized, the sticky malodor was instead attributed to “mold houses” [27,36]. These “mold houses” were not described in the scientific domain. As Sweden became the world leader in indoor air research [26], spurred by the continuous malodor and coinciding health complaints [12], the malodorous CAs and toxic CPs were not mentioned. Instead, they became a silent part of various environmental factors in a new mindset and terminology that evolved in the early 1980s, with malodor being central, such as “sick houses” where people developed “sick building syndrome” (SBS), asthma, and allergy [12]. Even after 1999, when commercial analyses of CPs and CAs in indoor air had become available in Sweden [28] and known among building investigators [12,25,27], these problematic chemicals were still not mentioned in the scientific domain. Obviously, alternative sources of information must therefore be used to determine the impact of CPs and CAs on housing and health and why they were not mentioned in the scientific literature.

Here, we scrutinize research conference contributions, parliament records, newspaper articles, and cartoons to address four basic questions: (1) when did the term “mold houses” appear?; (2) how did the “mold houses” impact people and society?; (3) were SBS researchers aware of the CAs?; and if so, (4) why did they not mention them in their papers?

## 2. Materials and Methods

### 2.1. Mentioning of “Mold Houses” in Newspapers, Cartoons, and the Parliament

When did the term “mold houses” appear and how did the “mold houses” impact people and society? Using the Swedish word for “mold houses” (i.e., “mögelhus”), we searched for records of any forms of the word, using the wild card symbol * (mögelhus*) in three different information sources. First, the digitalized newspaper archive at The National Library of Sweden was searched using the public website search function [37] and identified newspaper articles were obtained from the library. Second, a hands-on search was made in the complete collection of yearly chronicles, from 1957 to 1988, of cartoons by Staffan Lindén. The cartoons, called Staffans Stollar, captured contemporary societal talking points in singlet comical drawings, and were regularly published in newspapers, magazines, etc. [38]. Third, the Swedish Parliament website was searched, and hits (records) were sorted by date using the public search and sorting functions [39]. In the retrieved records on “mold houses” from the three different sources, we primarily searched for the following information: when the term was used, odor descriptions, mentioning of preserved wood, effects of the odor on people, how many “mold houses” there were, and whether they warranted societal actions. Some relevant and illustrative quotes were reproduced in English in the Results section. Concerning parliament records, original Swedish records and text is given in Supplement S1, as well as the English translations of quotes, marked in italics.

### 2.2. Mentioning of CAs by SBS Researchers Outside Peer-Reviewed Journals

Did Swedish SBS researchers know of CAs, and if so, why did they not mention this in peer-reviewed journals? We reasoned that when the company Pegasuslab started marketing measurements of CAs and CPs, in 1999 [28], this would lead to questions for SBS researchers considering that the problematic chemicals were not yet recognized in buildings. Thus, some communication between researchers might have occurred at indoor air conferences. We used a two-step procedure to search for such potential mentioning of CAs by Swedish SBS researchers in conference proceedings. First, we identified Swedish SBS researchers by searching the Web of Science Core Collection database for the term “sick building syndrome” (including parentheses) in All Fields, choosing a Publication Date from 1 January 1945 to 31 August 2024, and refining with Article and Review Article in the Document Types filter. The results were analyzed by Authors, Country/Region, and Affiliations. For Authors, Show Researcher Profiles was disabled and manual curation was made for various spellings of the same author name, such as full first name or initial(s) and use of Nordic letters (å, ä, ö, ø). Second, we searched for any mentioning of CAs by the identified Swedish SBS authors by reading through the proceedings of two triannual conferences, namely the Swedish Indoor Climate conferences organized by the Occupational and Environmental Medicine clinic in Örebro, Sweden, and the Indoor Air conferences held in different countries by the International Society of Indoor Air Quality and Climate (ISIAQ). The searches in proceedings were restricted to the period between 1999, when measurements of CAs were first marketed [28], and 2016, when we were first to report CAs in a peer-reviewed journal [25].

## 3. Results

### 3.1. “Mold Houses” in Swedish Newspapers

**Figure 2:**
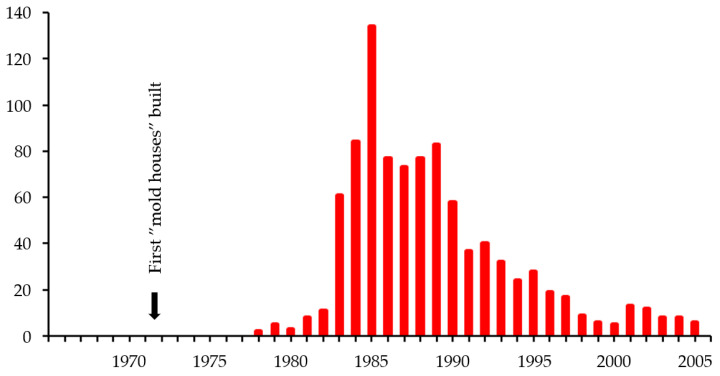
Number of newspaper articles mentioning “mold houses” in 1965–2006. The arrow points to 1971–1972 when the “mold houses” were built in the first articles mentioning “mold houses” in 1978–1980.

“Mold houses” first appeared in newspapers in 1978. The number of articles per year increased rapidly (Figure 2), with more than 600 articles published in the 1980s mentioning “mold houses”. We chose to focus on the earliest articles, published in 1978–1980, i.e., before the term reached the Swedish parliament. Nine articles were published in this period [40,41,42,43,44,45,46,47,48], whereof one only mentions “mold house” in passing [43]. The remaining eight articles address “mold houses” in depth. In all cases the odor is described as equating mold. Five articles specify when the “mold house” was built, 1971 to 1973 [40,44,45,46].

The first article, from 1978, describes a sticky odor in two community houses for children, built in 1972, and the extensive attempts to get rid of the odor. The title is telling: ‘The mold house-it may have to be demolished’ [40]. Note that all quotes herein were translated from Swedish by us. We note that experts from Chalmers university in Gothenburg featured in tree of the first four articles [40,42,44], two of which cite John Eriksson, professor of mycology [42,44]. He stated that there were thousands of “mold houses” [42]. Five articles describe them as costly to remediate [40,42,44,45,48]. They were difficult to sell [46] and could lead to economic ruin [48]. All eight articles describe uncertainty as to the causes, seven refer to legal problems and disputes [40,41,42,44,45,46,48]. Eriksson and his fellow researchers opined that the “mold houses” were caused by faults and carelessness during the building phase, leading to dampness, mold, and odor [42]. Seven articles mention odor [40,41,42,44,46,47,48], whereof six describe it as sticky [40,41,42,44,46,48]. One article describes the hardships of a family as their new dream house started to smell and turned into a two-year long nightmare [44]. Everything smelt of mold, including clothes, furniture, and curtains. The family could not socialize because of the smell. The mother stated about her son that ‘Johan never smelled like a baby; he smelled like mold’. The father had found the odor source to be impregnated wood cast into concrete in the house foundation [44]. One more article points to preservatives, stating that it is easy to know if there is mold in the house, as it smells and can be found in the sill plates, even though they are impregnated [48]. Four articles describe health issues, asthma [46], allergy [41,45,46], psychological impact [46,48], and a sick baby [41]. In one case, concerning 15 new houses in a neighborhood, the headline was particularly alarming: ‘Mold attack on houses in Bårslöv, the owners are forced to stay, 17 persons sick’ [46]. In the following years, most articles mentioning “mold houses” were published in 1985 (Figure 2). Of the 134 articles published in 1985, five were published the same day and all dealt with the state television announcing potential help to owners of “mold houses” through a “mold house” fund [49,50,51,52,53]. Later that same year, the “mold house” fund was decided, and a newspaper reported that rescue had arrived: ‘End of economic ruin, end of processes and disputes, end of sitting between involved holders of power, with no one wanting to take responsibility. Now it’s finally over’ [54]. This expectation did not come true, partly because the government agencies did not recognize the CP problem [12,25,26].

### 3.2. “Mold Houses” in Swedish Cartoons

**Figure 3:**
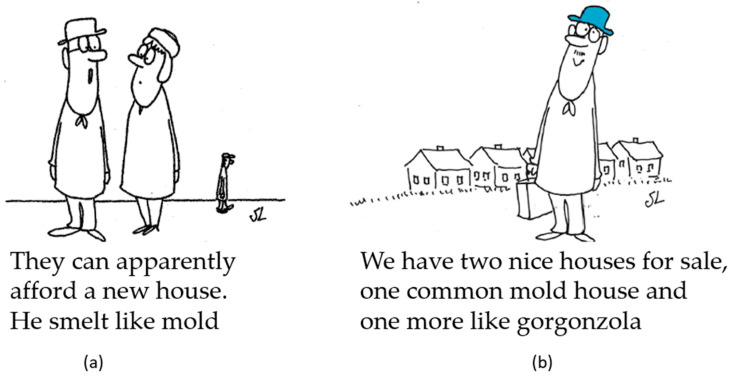
Cartoons that capture typical aspects of CAs in buildings. (**a**) The typical sticky malodor. (**b**) The term “mold house”. Reproduced with permission by Mats Lindén (son of the artist). The text was translated from Swedish by us.

A Staffans Stollar cartoon from 1984 [55] refers to the typical sticky odor, showing a man commenting on someone passing by: ‘They can apparently afford a new house. He smelt like mold’ (Figure 3a). The term “mold houses” appeared one time, in 1988 [56]. The cartoon shows a house broker saying the following: ‘We have two nice houses for sale, one common mold house and one more like gorgonzola’ (Figure 3b).

The figures were reproduced with permission by Mats Lindén (son of the artist). The text was translated from Swedish by us.

### 3.3. “Mold Houses” in Swedish Parliament Documents

#### 3.3.1. Retrieved Records of Mold Houses

**Figure 4:**
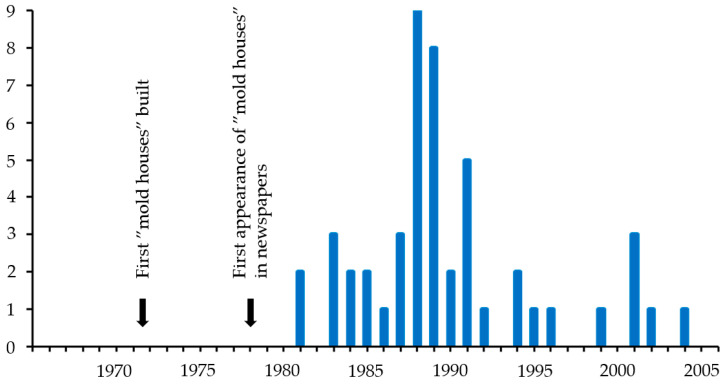
Number of parliament records mentioning “mold houses” in 1965–2006. The left arrow points to 1971–1973 when the “mold houses” were built in the first newspaper articles mentioning “mold houses” in 1978–1980. The right arrow points to 1978 when “mold houses” first appeared in newspaper articles.

Altogether, 50 records covering 27 years were retrieved and listed in chronological order between 1981 and 2007 (No. 1–50) (see Supplement S1). They were of five different types: Reports and Statements (*n* = 8), Interpellations (*n* = 1), Chamber protocols (*n* = 23), Members motions (*n* = 15), Government bills and Written communications (*n* = 1), and Swedish Government Official Reports/reports by Government commissions of inquiry (SOU series) (*n* = 2). Most records, 30/46, were from the first nine years, with the highest numbers in 1988–1989 (Figure 4).

#### 3.3.2. Descriptions of Mold Odor and Its Origin in Buildings, Including Preserved Wood

Detailed descriptions of sticky odor are given in the two first records from the Parliament, both from 1981. The second record is illustrative (No. 2): ‘For the affected families, the inconvenience is often very great. It is not only the financial problems I think about but also the mental ones because the unpleasant odor that gets stuck in clothes, hair and furniture leads to, for example children being bullied at school, and the family is reluctant to invite guests to their home because of the smell etc.’ Further, ‘Another complication is that mold infestation occurs even when current standards have been followed and when construction cheating has not been detected’.

The first record also mentions preserved wood (No. 1): ‘Most often it is the frame of the house, framing of joists, and floors that are attacked. For various reasons, the humidity is too high in the said parts which over time leads to the appearance of mold fungus that spreads a very unpleasant smell that gets stuck in clothes, hair, furniture, carpets etc. The psychological discomfort can in many cases be large.’ Further, ‘The causes of mold are many and there is great uncertainty as to the main cause in each individual case. Moist ground, houses built in winter, too dense or incorrect insulation, and pressure-treated timber in the inner structure.’

Later, at a public inquiry in 1988 (No. 22), Ingemar Samuelson (in our opinion, the leading expert on malodorous houses) also points to treated wood as an odor source: ‘Sills cast in concrete are a suitable way to build quickly from a production point of view. But we now know that even if the sills are pressure-impregnated, you get mold growth and odor from them.’ Further, ‘most newly built houses are both drier and better than older houses, but they smell’.

#### 3.3.3. Societal Actions and Investigations

The second record mentions that in 1978 The National Swedish Institute for Building Research started a project aimed at finding the reasons for mold attack. The estimated number of “mold houses” were 18,000 (No. 2). Most of the early records deal with, or mention, the financial perils of afflicted house owners. This led to political action. One record, from 1987, states that ‘The reason why the parliament decided to form a fund for dampness and mold damages was that it was complicated to determine responsibility, so that people afflicted by a mold house can get help promptly’. (No. 14). Most of the records in 1988–1989 (Figure 1) deal with issues regarding this economic compensation from the state. We note that three records were retrieved due to the term “dampness- and mold house”, rather than “mold house” (No. 37, 38, 43); they all appeared after the state “Dampness and mold fund” was created. Several records from 1988 to 1989 also cover health issues, for example, a public inquiry in 1988 on “sick houses” (No. 22) where Samuelsson explains, ‘What do we mean by sick houses? Common concerns in such houses are ill health and odor.’ Another expert estimates the number of affected homes to be unknown, but at least 10,000, and refers to two investigations: the recently completed ‘Healthy and sick houses’, and an ongoing ‘Allergy investigation’ (No. 22). This latter investigation is also referred to in a record from 1989 (No. 26): ‘The increasing problems of allergy sufferers are highlighted in a special investigation, among other things. We have demanded increased efforts for the remediation of so-called mold houses in another motion.’ Five records mention allergies (No. 6, 19, 22, 26, 34).

With time, “mold houses” became synonymous with bad odor and impropriety, for example: ‘Sweden may look calm and prosperous on the surface. In fact, parts of our legal system and our exercise of power are rotting from within. Sweden is like a mold house. It doesn’t smell good’. (No. 27). Further, ‘Recently, a number of outrageous cases were documented of cheating and carelessness being the standard—mold houses, dilapidated bridges and asphalt cartels’. (No. 46, 47), and ‘we stand at what we call a scandal, the doctors that dismember bodies, priests that rape boys, bankers that cheat, mold houses, etc.’ (No. 48).

### 3.4. Swedish SBS Researchers and Mentions of CAs at Conferences

#### 3.4.1. SBS Researchers

Our Web of Science search on SBS yielded 1417 articles. Divided by country, USA is most productive (349), and Sweden is second (181). Notably, Sweden and the neighboring Nordic countries, Denmark (119), Finland (67), and Norway (35), all with small populations, account for more articles combined (363) than the more than tenfold more populous USA. By affiliation, most Swedish articles were produced in cities with university hospitals, with the most productive city being Uppsala, followed by Stockholm. Uppsala University/Hospital is even the most productive affiliation globally. Comparing authors world-wide, Sweden occupies the top three positions in publication output, but many other Swedes contributed to the SBS field as well, as will be described in the following section.

#### 3.4.2. Awareness of CAs at Conferences by SBS Researchers

We found no verbatim mentions of CAs by Swedish SBS researchers, but two conference contributions show awareness [57,58]. In 2000, at the Indoor Climate conference in Örebro, Göran Stridh dealt with the utility of indoor air measurements. This included chemicals that could signal problems in buildings [57]. Table 3, in the presented conference paper, contains impregnated wood and informs that the signal substances are “several, mainly anisoles” [57]. The stated reason for the anisoles is dampness. Further, Stridh made it clear that measurements can be used to show technical faults in buildings and not to make statements about health effects. Eight years later, at the 2008 international Indoor Air conference in Denmark, two authors from Pegasuslab in Uppsala concluded from their results, on creosote, CPs, and CAs, that there is a clear correlation between complaints of bad indoor air quality and presence of chloroanisoles in indoor air [58]. They suggested that more attention to wood preservative chemicals in building constructions is needed. The authors contrasted this to the statement: “The general consensus among field investigators and medical expertise (M.D. Gunilla Wieslander, Occupational Medicine, University Hospital of Uppsala, 2007, pers. comm.) is that wood preservatives only constitute an odor problem and are not connected to health complaints”. [58].

## 4. Discussion

First, we asked the following: when did the term “mold houses” appear? We show that the term first appeared in Swedish newspapers in 1978. Notably, the first described “mold houses” were built in 1971–1973, in some, a stench evolved almost immediately. The sticky malodor was typical; it is well captured in an article from 1979 where a mother says that her son never smelled like a baby, he smelled like mold. Several articles describe allergy, asthma, and psychological impact. Some point to impregnated wood as the odor source. With this new information, we can build an interesting timeline. As previously reported [12], an unfamiliar sticky odor spread nationwide in the early 1970s, giving rise to many newspaper articles, including one from 1973 with the illustrative title: ‘The school stinks but nobody knows why’ [59]. A local investigator suspected impregnated wood, but he changed his opinion in a follow-up paper: ‘Mold-that is why the school stinks’ [60]. Experts at the nearby Chalmers University had expected the suspected floor construction to be rotten; instead, it was fresh but contained mold [60]. We have now found that Chalmers also featured in the first newspaper articles on “mold houses” and claimed that “mold houses” were caused by building faults and carelessness during house construction leading to moisture. We have previously reported that the houses had been built according to the existing norms and regulations, leading to moisture in certain parts of the constructions, requiring wood preservatives to avoid wood rot [12]. Our searches now show that Chalmers was key in forming the narrative whereby the unfamiliar sticky odor became “mold odor” in 1973, and the afflicted houses became “mold houses” in 1978.

Second, we asked the following: how did the “mold houses” impact the Swedish society? We found a rapid increase in the number of newspaper articles on “mold houses” in the early 1980s, as well as cartoons on the subject. Furthermore, we found that the term “mold houses” appeared in the Swedish parliament already in 1981. The earliest records contain long descriptions of the sticky odor causing psychosocial problems, with many thousands of families being afflicted, faced with high costs for remediation and accountability disputes. The state established a fund to provide financial help to private house owners. However, other types of owners and afflicted buildings were not eligible.

All the publicity and attention are in line with our previous highlighting [12] of two contemporary reports by Samuelson [61,62], issued in 1981 and 1985 by the National Testing Institute, where he describes the houses with sticky odor in depth; see our appended translations [12]. Clearly, the sticky odor in the Samuelson reports, ‘Mold odor in houses’ and ’Mold in houses’, is the same as in the “mold houses” described in newspapers and parliament records. Furthermore, Samuelson instead used the English term “sick houses” at the 1984 international indoor air conference in Stockholm [63]. “Sick houses” was a new term, and we interpret Samuelson’s conference contribution to be a message to other countries that Sweden was going to handle houses with sticky odor as being caused by mold growth in damp locations. Thomas Lindvall, one of the conference organizers, was key to this development. He was a Swedish government agency representative in the WHO working group that established SBS in 1983 [64], and he co-founded ISIAQ and the Indoor Air journal [26].

We show that in the parliament, several records point to impregnated wood as an odor source, for example, Samuelson mentioned this at a hearing in 1988. He also said that odor and ill health is typical of “sick buildings”. Several parliament records mention allergies. The numbers of records mentioning “mold houses” were highest in 1988–1989, coinciding with a commissioned ‘Allergy investigation’. As previously reported, leading SBS researchers used the term “mold houses” in this investigation [27], including Sundell, former government agency official, PhD student of Lindvall, and a longtime chief editor of the Indoor Air journal [26].

Third, we asked the following: did Swedish SBS researchers know of CAs? We show that both newspapers and parliament records point to impregnated wood as an odor source in “mold houses”. This new information corroborates the reason given by the Swedish Wood Protection Institute for publishing its 1994 report on ‘Odor from impregnated wood’, namely that there were founded common suspicions against preservatives, but it was uncalled for to introduce restrictions because the sticky malodor in “mold houses” was due to an already abandoned preservative containing CPs [36]. The report gave an example of a neighborhood with 36 houses built in 1972–1973, where sticky malodor developed within a few years, and allergies and hypersensitivities were common. Taken together, it seems highly unlikely that Swedish world-leading indoor air researchers were unaware of these circumstances. It is even more unlikely that they remained unaware after Pegasuslab reported, in 1999, that CAs evoked sticky mold odor [28]. Indeed, we found evidence that SBS researchers were aware of the CAs. Our search in Web of Science confirmed our suspicion. Several Swedish researchers contributed to the SBS field; three of them were even the most productive amongst all authors world-wide. When searching for the Swedish SBS authors in meeting proceedings, we found that Stridh knew of CAs in 2000 [57]. He co-organized a series of Indoor Climate conferences in Örebro with Kjell Andersson and presented CAs at the meeting in 2000 [57]. At the time, CAs had only been reported indoors by Pegasuslab, in 1999. Notably, Stridh used the word anisoles, rather than CAs (leaving out “chloro”), and he did not mention CPs. Furthermore, Stridh presented the anisoles as indicating wood impregnation, and did not mention mold. Our second finding of an SBS researcher being aware of CAs is a 2008 article by authors from Pegasuslab, referencing personal communication with Wieslander on “wood preservative odor” [58]. In summary, we provide strong evidence that Swedish SBS researchers were aware of CAs and knew that they evoked malodor that was described as mold odor by Pegasuslab and known as such in the society.

Fourth, we asked why did Swedish SBS researchers not mention CAs in peer-reviewed journals? We show that Stridh opined in 2000 that air measurements, including anisoles, can only be used to show technical faults in buildings and not to make statements about health effects. It is relevant to point out that Stridh followed this up in Swedish periodicals of the building sector, by publishing several papers with the same message, but leaving out anisoles. One of his co-authors in these articles was Samuelson [65,66], the main national expert on field investigations [61,62,63]. Their opinion is in line with our finding of a 2008 quote of Wieslander stating that it was consensus among field investigators and medical expertise that wood preservatives only constitute an odor problem and are not connected to health complaints. Taken together, it becomes clear that the CAs were not mentioned in peer-reviewed articles because they were considered irrelevant in relation to health. This disregard was seemingly performed without any exposure and risk assessments of the CPs despite their known use. Furthermore, the SBS researchers decided that CAs evoked wood preservative odor but not mold odor, and therefore, in society and the scientific literature, mold odor continued to be an indicator of alleged hazardous hidden mold.

To our knowledge, the findings presented herein have not previously been published in the academic domain. Thus, our study is the first that describes the impact of an indoor odor, not only on a few people, but on society. It explains how mold became a permanent scapegoat. We provide answers to four questions that add important new dimensions to the lack and lag of scientific reporting on CAs in houses for humans, namely that the malodorous CAs gave rise to many problematic buildings called “mold houses” which provided major and long-lasting impetus for indoor air research and became known internationally as “sick buildings”. Furthermore, the CAs were known by researchers, yet made invisible in their peer-reviewed articles, just like the malodorous impregnated wood, and the CPs in wood preservatives. Consequently, the malodorous CAs and toxic CPs were not mentioned in a historical account of international indoor air research dating back to the 1960s, published by Sundell in Indoor Air 2017 [67]. Meanwhile, mold odor continued to be used in epidemiological studies as an important indicator of alleged hazardous hidden mold [68,69]. It follows that much research is seriously confounded, for example, when SBS and allergy is attributed to mold, “dampness and mold”, or simply dampness.

Concerning formation of mold odor today, CAs still evolve in buildings with legacy wood preservatives (there are no other sources of CA formation than CPs). However, the levels of CAs as well as those of CPs are generally in the order of a few ng/m^3^ in indoor air [25] and, in the case of the most odor potent CA congener 2,4,6-trichloroanisole, close to the odor detection limit, i.e., below 13 ng/m^3^ [12]. Our toxicological evaluation suggests that the CPs and CAs are not detrimental to human health at these concentrations [25], but sufficiently high to cause malodor [12,25]. While the malodorous Swedish “mold houses” lacked visible mold, even when opening the building constructions, it is conceivable that odor can develop in cases of major mold growth. The malodor would most likely be due to a complex and variable mixture of microbial volatile organic compounds at low levels [25]. To the best of our knowledge, no microbial volatile(s) underlying large scale mold odor has been reported, besides CAs. Moreover, it is easy for a trained nose to detect CAs among other potential indoor malodors. Indeed, it is common among Swedish building investigators to train odor recognition using 2,4,6-trichloroanisole and other odorants provided by Pegasuslab, since 1999 [28]. In many cases, this recognition guides further building investigations and assessments of indoor air quality (IAQ).

In future research, it would be interesting to further investigate the circumstances around the unique Swedish ban of CP in 1977, and why the authorities failed to inform the public about the presence and potential health hazards of CPs in building and the related formation of the malodorous CAs. This failure is highly remarkable as Sweden had world-leading expertise on CPs [35], and acute and chronic poisonings due to the use of CPs indoors, as well as in workplaces, were described at the time [2,3,4,5].

## 5. Conclusions

Mycologists at Chalmers university in Gothenburg were key players in forming a narrative whereby the indoor sticky CAs became “mold odor” in 1973, and the malodorous houses became “mold houses” in 1978. The term first appeared in newspapers in 1978 and reached the Swedish parliament in 1981. Several hundred newspaper articles addressed “mold houses” in the 1980s and the topic even became the subject of cartoons. The parliament dealt with various issues, such as how to help owners afflicted by the sticky malodor that no one took responsibility for. A state fund was established for this purpose. Impregnated wood was suspected in both newspaper articles and the parliament. Allergies occurred in the “mold houses”, giving rise to an ‘Allergy investigation’ warranting more research. With time, the “mold houses” were less often mentioned. They became “sick buildings” as Swedish researchers became world leaders on SBS. Researchers were aware of CAs but considered them to indicate wood preservatives, not mold, and to be of no relevance for health, and therefore not worth mentioning in scientific articles. Meanwhile, mold odor continued to be used in the scientific literature as an indicator of alleged hazardous hidden mold in house constructions. Thus, the mold story from the early 1970s prevented the malodorous CAs from becoming recognized as indicators of hazardous CPs and key players in research on IAQ, SBS, asthma, allergy, “sick houses”, “dampness and mold”, etc.

## Data Availability

The original contributions presented in this study are included in the article/supplementary material. Further inquiries can be directed to the corresponding author.

## Supplementary Materials

The following supporting information can be downloaded at: https://www.mdpi.com/article/10.3390/microorganisms13061363/s1, File S1: Retrieved Swedish parliament records mentioning “mold houses”.

## References

[1] Bevenue A., Beckman H. (1967). Pentachlorophenol: A discussion of its properties and its occurrence as a residue in human and animal tissues. Residue Rev..

[2] Crosby D.G. (1981). Environmental chemistry of pentachlorophenol. Pure Appl. Chem..

[3] WHO, IPCS (1987). Environmental health criteria 71. Pentachlorophenol.

[4] WHO, IPCS (1989). Environmental Health Criteria 93. Chlorophenols Other Than Pentachlorophenol.

[5] Jorens P.G., Schepens P.J. (1993). Human pentachlorophenol poisoning. Hum. Exp. Toxicol..

[6] Eisler R. (1989). Pentachlorophenol hazards to fish, wildlife, and invertebrates: A synoptic review. Biological Report.

[7] IARC (2019). Carcinogenicity of Pentachlorophenol and Some Related Compounds, IARC Monographs on the Evaluation of Carcinogenic Risks to Humans.

[8] Stockholm Convention. All POPs Listed in the Stockholm Convention. Annex A (Elimination). https://www.pops.int/TheConvention/ThePOPs/AllPOPs/tabid/2509/Default.aspx.

[9] Ide A., Sakamoto F., Watanabe H., Watanabe I., Niki Y. (1972). Decomposition of Pentachlorophenol in Paddy Soil. Agric. Biol. Chem..

[10] Lee H.B. (1988). Determination of twenty-one chloroanisoles in water and sediment samples. J. Assoc. Off. Anal. Chem..

[11] Glickman A.H., Statham C.N., Wu A., Lech J.J. (1977). Studies on the uptake, metabolism, and disposition of pentachlorophenol and pentachloroanisole in rainbow trout. Toxicol. Appl. Pharmacol..

[12] Lorentzen J.C., Juran S.A., Ernstgard L., Olsson M.J., Johanson G. (2020). Chloroanisoles and chlorophenols explain mold odor but their impact on the Swedish population is attributed to dampness and mold. Int. J. Environ. Res. Public Health.

[13] Whitfield F.B., Nguyen T.L., Shaw K.J., Last J.H., Tindale C.R., Stanley G. (1985). Contamination of dried fruit by 2,4,6-trichloroanisole and 2,3,4,6-tetrachloroanisole adsorbed from packaging materials. Chem. Ind..

[14] Buser H.R., Zanier C., Tanner H. (1982). Identification of 2,4,6-trichloroanisole as a potent compound causing cork taint in wine. J. Agric. Food Chem..

[15] Saxby M.J., Wragg S. (1985). Contamination of cocoa liquor by chlorophenols. Chem. Ind..

[16] Spadone J.C., Takeoka G., Liardon R. (1990). Analytical investigation of rio off-flavor in green coffee. J. Agric. Food Chem..

[17] Nyström A., Sävenhed R., Krantz-Rüilcker C., Grimvall A., Åkerstrand K. (1992). Drinking water off-flavour caused by 2,4,6-trichloroanisole. Water Sci. Technol..

[18] Bemelmans J.M.H., Ten Noever De Brauw M.C. (1974). The presence of chloroanisoles in tainted tobacco. Sci. Total Environ..

[19] Ramstad T., Walker J.S. (1992). Investigation of musty odor in pharmaceutical products by dynamic headspace gas-chromatography. Analyst.

[20] Hill J.L., Hocking A.D., Whitfield F.B. (1995). The role of fungi in the production of chloroanisoles in general-purpose freight containers. Food Chem..

[21] Engel C., de Groot A.P., Weurman C. (1966). Tetrachloroanisol: A source of musty taste in eggs and broilers. Science.

[22] Curtis R.F., Land D.G., Robinson D., Gee M., Gee J.M., Griffiths N.M., Peel J.L., Dennis C. (1972). 2,3,4,6-Tetrachloroanisole association with musty taint in chickens and microbiological formation. Nature.

[23] Dennis C., Gee J.M. (1973). The microbial flora of broiler-house litter and dust. J. Gen. Microbiol..

[24] Curtis F., Dennis C., Gee J.M., Gee M.G., Griffiths N.M., Land D.G., Peel J.L., Robinson D. (1974). Chloroanisoles as a cause of musty taint in chickens and their microbiological formation from chlorophenols in broiler house litters. J. Sci. Food Agric..

[25] Lorentzen J.C., Juran S.A., Nilsson M., Nordin S., Johanson G. (2016). Chloroanisoles may explain mold odor and represent a major indoor environment problem in Sweden. Indoor Air.

[26] Lorentzen J.C., Harderup L.E., Johanson G. (2023). Evidence of unrecognized indoor exposure to toxic chlorophenols and odorous chloroanisoles in Denmark, Finland, and Norway. Indoor Air.

[27] Lorentzen J.C., Ekberg O., Alm M., Bjork F., Harderup L.E., Johanson G. (2024). Mold odor from wood treated with chlorophenols despite mold growth that can only be seen using a microscope. Microorganisms.

[28] (1999). Mögellukt är inte alltid mögel.

[29] Palm H., Knuutinen J., Haimi J., Salminen J., Huhta V. (1991). Methylation products of chlorophenols, catechols and hydroquinones in soil and earthworms of sawmill environments. Chemosphere.

[30] Haimi J., Salminen J., Huhta V., Knuutinen J., Palm H. (1993). Chloroanisoles in soils and earthworms. Sci. Total Environ..

[31] Renberg L., Marell E., Sundstrom G., Adolfssonerici M. (1983). Levels of chlorophenols in natural-waters and fish after an accidental discharge of a wood-impregnating solution. Ambio.

[32] Bertrand A., Barrios M.L. (1994). Contamination des bouchons sur les produits de traitments de palletes de stockage des bouchons. Rev. Fr. Oenol..

[33] Chatonnet P., Guimberteau G., Dubourdieu D., Boidron J.N. (1994). Nature et origine des odeurs de “moisi” dans les caves. Incidences sur la contamination des vins. J. Int. Sci. Vigne Vin.

[34] Gunschera J., Fuhrmann F., Salthammer T., Schulze A., Uhde E. (2004). Formation and emission of chloroanisoles as indoor pollutants. Environ. Sci. Pollut. Res. Int..

[35] Ahlborg U.G., Thunberg T.M., Spencer H.C. (1980). Chlorinated phenols: Occurrence, toxicity, metabolism, and environmental impact. Crit. Rev. Toxicol..

[36] Nyman E. (1994). Lukt Från Impregnerat Trä.

[37] Sök i Svenska Tidningar (National Library of Sweden Search Function). https://tidningar.kb.se/.

[38] Konstnärslexikonett Amanda. https://www.lexikonettamanda.se/show.php?aid=19573.

[39] Sveriges Riksdag, Sök (Swedish Parliament Search Function). https://www.riksdagen.se/sv/sok/?avd=dokument.

[40] Magnusson E. (1978). Mögelhuset. Nu kanske det måste rivas.

[41] (1978). Snabbåtgärder utlovade för mögelhusen i Vegby.

[42] (1979). Forskare till attack—Byggslarvet vållar dyra mögelskadorna.

[43] (1979). Rinmans elevkår vill skapa ungdomens hus—nu ska lokalerna bort.

[44] Arthursson O. (1979). Familjen Stark, några av tusentals offer för mögelangreppen.

[45] Ringberg L. (1979). Huset felbyggt—nu kräver familjen ersättning.

[46] Schwanbom G. (1979). Mögelangrepp på hus i Bårslöv—ägarna tvingas bo kvar—17 Personer Sjuka.

[47] Håård L. (1980). Det luktade mögel—hur man än städade.

[48] (1980). Isolerat, varmt, men mögelhus.

[49] (1985). Utredning synar mögelhus.

[50] (1985). Ny utredning om mögelhus.

[51] (1985). Mögel utreds.

[52] (1985). Fond för mögelhus skall snabbt utredas.

[53] Wester M. (1985). Fond för mögelhus regeringsförslag.

[54] (1985). Räddningen för mögelhusdrabbade.

[55] Lindén S. (1984). Staffans Stollar: En Årskrönika i Bild 1984.

[56] Lindén S. (1988). Staffans Stollar 88.

[57] Stridh G. Vilka slutsatser kan dra av kemiska mätningar?. Proceedings of the Inomhusklimat.

[58] Wessén B., Lager J. The correlation of microbial and chemical contents of indoor air and percieved IAQ, Paper ID: 104. Proceedings of the Indoor Air, 11th International Conference on Indoor Air Quality and Climate.

[59] Ramqvist I. (1973). Det stinker i skolan—Men Ingen vet Varför.

[60] Zetterström Å. (1973). Mögel—Därför luktar skolan pyton.

[61] Samuelson I. (1981). Mögelluktande Hus. Redovisning av Skadefall.

[62] Samuelson I. (1985). Mögel i Hus. Orsaker och Åtgärder.

[63] Samuelson I. Sick houses—A problem of moisture?. Proceedings of the Indoor Air: The 3rd International Conference on Indoor Air Quality and Climate.

[64] WHO (1983). Indoor Air Pollutants: Exposure and Health Effects.

[65] Andersson K., Stridh G., Ekberg L., Samuelson I. (2004). Kemiska, mikrobiologiska och partikelmätningar—Hjälpmedel eller “big business”?. Bygg Tek..

[66] Andersson K., Stridh G., Ekberg L., Samuelson I. (2005). Se till att få det du betalar för!. Bygg Tek..

[67] Sundell J. (2017). Reflections on the history of indoor air science, focusing on the last 50 years. Indoor Air.

[68] WHO (2009). WHO Guidelines for Indoor Air Quality: Dampness and Mould.

[69] Mendell M.J., Kumagai K. (2017). Observation-based metrics for residential dampness and mold with dose-response relationships to health: A review. Indoor Air.

