# Supplementary material for: Evidence of Malodorous Chloroanisoles in “Mold Houses” Was Omitted When Indoor Air Research Evolved
Source: Microorganisms. 2025 Jun 12;13(6):1363. doi: 10.3390/microorganisms13061363 (PMC12196426; doi:10.3390/microorganisms13061363)
Supplement: Supplementary file 1 [file microorganisms-13-01363-s001.zip › microorganisms-3682483-SM.pdf]

Evidence of malodorous chloroanisoles in "mold houses" was omitted when indoor air research evolved

Johnny C. Lorentzen and Gunnar Johanson

File S1. Retrieved Swedish parliament records mentioning "mold houses".

Using the public search function on the website of the Swedish Parliament we searched for records mentioning any forms of the Swedish word för "mold house" (i.e., "mögelhus") using the wild card symbol * (i.e., mögelhus*). The search was performed 10th of September 2024 and resulted in 50 hits (records) as listed below between 1981 and 2007, together with key original Swedish text pieces that were used in our analyses, and with the search term ("mögelhus*") marked in bold. For each record, the text piece presented by the website is given first, in some cases complemented with additional text selected after reading through the complete record. Sentences that were translated to English and quoted in the article are marked in italics.

1. Motion 1980/81:834 av Bertil Danielsson m. fl
https://www.riksdagen.se/sv/dokument-och-lagar/dokument/motion/om-fukt-och-mogelskador-i-byggnader_g402834/

… Denna har emellertid upphört på grund av aviserade kraftiga premiehöjningar. Således kan konstateras att för flertalet **mögelhus** finns i dag ingen försäkring. Eftersom det i de flesta fall är relativt nybyggda hus som drabbas, är boendekostnaderna redan så höga att ytterligare kostnader inte är möjliga att klara av. Mot. 1980/81:834 6 Genom riksdagsbeslut 1980 finns det möjlighet att få s. k. radonlån till ombyggnad av hus med för hög radonstrålning. …

Oftast är det husets stomme, bjälklag och golv som angrips. Av olika skäl är fuktigheten för stor i de sa delar_ vilket med tiden leder till att mögelsvamp uppträder. Denna sprider en mycket obehaglig lukt som sätter sig fast i kläder, hår, möbler, mattor o.s.v. Det psykiska obehaget kan i många fall vara stort. Exempelvis uppges att barn som bott i s.k. **mögelhus** blivit mobbade i skolan på grund av lukten.

Orsakerna till att mögel uppstår är många och osäkerheten stor beträffande huvudanledningen i varje enskilt fall. Några "standardsituationer" har av forskningen kunnat iakttas: hus byggda på cementplatta utan källare; sank, fuktig mark; hus byggda på vintern; alltför tät eller felaktig isolering och tryckimpregnerat virke i innerkonstruktionen.

Kostnaderna för att komma till rätta med "**mögelhusen"** kan komma att uppgå till långt större belopp än för "radonhusen". Om staten skulle gå in med ekonomiskt stöd får detta i så fall en betydande omfattning. Med hänvisning till vad som anförts hemställs
1. att riksdagen hos regeringen hemställer att forskningen om fukt- och mögelskador i byggnader intensifieras och att en bred information om dess resultat genomförs,
2. att riksdagen hos regeringen hemställer om överväganden om möjliga åtgärder i syfte att stödja särskilt svårt drabbade ägare till mögelangripna hus.

2. Riksdagens protokoll 1980/81:118
https://www.riksdagen.se/sv/dokument-och-lagar/dokument/protokoll/riksdagens-protokoll-198081118-9-10-april-1981_g409118/

… Man har trots att rapporten inte har slutredovisats gjort vissa ändringar i anvisningar och normer för byggnadsverksamheten. Förhoppningsvis skall detta leda till att man kan förvänta sig allt färre mögelhus i framtiden. Men för den enskilde husägaren kan ett mögelangrepp få förödande verkningar. Endast ett fåtal småhus har försäkring som täcker mögelangrepp. Om skadorna är omfattande kan den enskilde husägarens ekonomi helt raseras. …

För de drabbade familjerna är ofta olägenheterna mycket stora. Det är inte bara de ekonomiska problemen jag tänker på utan även de psykiska, eftersom den obehagliga lukt som sätter sig fast i kläder, hår och möbler gör att exempelvis barn kan bli mobbade i skolan, familjen drar sig för att bjuda hem gäster på grund av lukten osv.

Förhoppningsvis skall detta leda till att man kan förvänta sig allt färre **mögelhus** i framtiden.
Men för den enskilde husägaren kan ett mögelangrepp få förödande verkningar. Endast ett fåtal småhus har försäkring som täcker mögelangrepp.

Jag har emellertid den uppfattningen att opinionstrycket för en lösning av problemen med **mögelhusen** ännu inte nått upp till det som föranleddes av radonhusen. Tiden och utvecklingen kommer nog att visa att detta inte är sista gången som frågan om **mögelhus** tas upp till behandling.

Fru talman! Ett något annorlunda problem tar jag tillsammans med Ewy Möller och Ingvar Eriksson upp i motion 834. Det gäller det allt större problemet med mögelangripna hus. Detta problem är ganska nytt. Särskilt markant har det blivit sedan byggande utan källare på betongplatta ökat. *En annan komplikation är att mögelangrepp uppstår även när gällande normer följts och när byggfusk inte kunnat konstateras.* Detta utesluter dock inte att fusk och slarv i en del fall kan vara orsaken
Vad som gör frågan särskilt svårhanterlig är att skadornas omfattning i en del fall är mycket stora. Saneringskostnaderna kan i särskilt svåra fall uppgå till 100 000 kr eller mera.

År 1978 igångsattes av byggforskningsrådet ett forskningsprojekt, där hittills ca 14 000 hus undersökts.

3. Riksdagens protokoll 1982/83:116
https://www.riksdagen.se/sv/dokument-och-lagar/dokument/protokoll/riksdagens-protokoll-198283116_g609116/

… Denna har emellertid upphört på grund av aviserade kraftiga premiehöjningar. Således kan konstateras att för flertalet **mögelhus** finns i dag ingen försäkring. Eftersom det i de flesta fall är relativt nybyggda hus som drabbas, är boendekostnaderna redan så höga att ytterligare kostnader inte är möjliga att klara av. Mot. 1980/81:834 6 Genom riksdagsbeslut 1980 finns det möjlighet att få s. k. radonlån till ombyggnad av hus med för hög radonstrålning. …

Men jag vill nog också säga att det är en mycket eländig situation som intet ont anande hus- och lägenhetsägare har försatt sig i när deras nyinköpta hus eller lägenheter - ofta deras livs investering – plötsligt visar sig vara **mögelhus**. Både hälsan och ekonomin rasar utför medan byggarna, myndigheter och konsulter träter om olika orsaker och om skuldfrågor.

4. Riksdagens protokoll 1983/84:5
https://www.riksdagen.se/sv/dokument-och-lagar/dokument/protokoll/riksdagens-protokoll-1983845_g7095/

… Är kommunikationsministern beredd att skjuta på detta ställningstagande tills Öresundsfrågan i sin helhet skall behandlas den 10 oktober 1983/84:52 av Gunnar Olssons till bostadsministern om mögel- och fuktskador i bostadshus. Det framstår med tydlighet att de personer som råkat illa ut i fråga om de s. k. **mögelhusen** är fler till antalet än vad man först trodde och att dessa hus medfört

betydande ekonomiska konsekvenser för dem som drabbats. En del av dem som förvärvat mögelskadade hus kan nu i efterhand konstatera att hela deras ekonomi rasar och att de står där med sitt mögelhusvars andrahandsvärde…

är väsentligt reducerat, där huset i många fall utpekas som omöjligt att bo i. Speciellt för personer med allergibesvär- och de är många i vårt land- har de här problemen fått rent förödande följder. Många har i samband med påfrestande processer och i kontakter med olika experter, byggherrar, advokater och försäkringsombud fått utstå mycket lidande.

5. Riksdagens protokoll 1983/84:8
https://www.riksdagen.se/sv/dokument-och-lagar/dokument/protokoll/riksdagens-protokoll-1983848_g7098/

… Regeringen borde hjälpa till så att vi snabbt kunde få ett prejudikat. Byggbolagen måste ta sitt ansvar och åtgärda dessa fuktskadade hus. Problemen med **mögelhusen** har under de senaste dagarna kommit att stå i fokus, bl. a. på grund av den uppmärksammade processen i Falun angående Falu kommuns bostadsområde Slätta med drygt 170 hus byggda under tiden 1979-1982, vilka har omfattande fukt- och mögelskador. …

Jag har med mig brev från fem av Värmlands kommuner, där drabbade människor beskriver sin situation. Jag skall bara citera ett par rader ur ett av breven som kommer från Kristinehamn: "Under psykisk press och med stor oro för hälsan har vi tvingats leva i ett mögelskadat hus i hela sex år. Sex års oförskyllt lidande måste få ett slut.

6. Motion 1983/84:461 Gunnar Olsson
https://www.riksdagen.se/sv/dokument-och-lagar/dokument/motion/mogel-och-fuktskador-i-hus_g702461/

… Hur skadorna skall betalas, hur möglet skall förebyggas och bekämpas och vilken roll trävirkets hantering från skogen till bygget har, därom tvista de lärde. **Mögelhusdebatten** rasar i oförminskad takt. Det framstår med tydlighet att de personer som råkat illa ut i fråga om de s. k. mögelhusen är fler till antalet än vad man först trodde och att det medfört betydande ekonomiska konsekvenser för dem som drabbats. …

En del som förvärvat mögelskadade hu kan nu i efterhand konstatera att hela deras ekonomi rasar och de står med sitt '"**mögelhus**". Vars andrahandsvärde är väsentligt reducerat, då det i många fall utpekas om omöjligt att bo i. Speciellt för personer med allergibesvär - och det är många i vårt land - har de här problemen också fått rent förödande följer. Många har i samband med påfrestande processer och kontakter med olika experter, byggherrar, advokater och försäkringsombud fått utstå mycket lidande. Uppskattningsvis rör det sig i hela landet om ca 100 000 fukt- och mögelskadade hus. Av dessa utgörs ca 75% av villor. Övriga utgörs av marklägenheter, daghem och skolor. Problemen finns över hela vårt land. Bara i Värmland har förekomsten av **mögelhus** i bl.a. Arvika, Kristinehamn, Säffle, Hammarö och Forshaga kommuner inneburit stora påfrestningar för olyckliga husägare.

7. Riksdagens protokoll 1984/85:30
https://www.riksdagen.se/sv/dokument-och-lagar/dokument/protokoll/riksdagens-protokoll-19848530_g80930/

… Jag vill nämna för Agne Hansson att ett av våra stora problem, som har större omfattning än radonproblemet och som i de flesta fall får minst lika allvarliga följder, är **mögelhusen**. De orsakar väldiga problem för människor som drabbas både psykiskt och fysiskt. Det är inte lätt, Agne Hansson,

att klara av alla uppkommande problem i världen med en gång. Vi gör så gott vi kan. När det gäller radonet har vi skapat finansieringsmöjligheter och vi har skapat normer. …

Det räcker inte med att konstatera att detta är ett allvarligt problem och att hänvisa till att vi har ett ännu allvarligare problem, nämligen mögelproblemet. Också jag vill livligt understryka att **mögelhusen** innebär ett stort problem. Men om regeringen nu är medveten om att problemen är så allvarliga, måste den försöka komma fram till konkret handling.

8. Motion 1984/85:1662 Karin Ahrland
https://www.riksdagen.se/sv/dokument-och-lagar/dokument/motion/oversyn-av-jordabalkens-bestammelser-om_g8021662/

… Säljaren kan i god tro ange att fastigheten har vissa egenskaper som senare visar sig inte vara för handen. De senaste årens tragedier till följd av bl. a. **mögelhusproblemen** väcker frågan vilket ansvar säljaren kan ha när hans hus är uppfört enligt gällande plan- och byggnadslagstiftning. Om isolering etc. är utförd enligt alla tillbörliga regler, är det då rimligt att det är säljaren som skall stå ansvaret…

De många fallen med s. k. **mögelhus** under senare år har ofta orsakat svåra tolkningsproblem av undersökningsplikten. För en vanlig husköpare torde det ofta vara omöjligt att göra en sådan teknisk besiktning av ett hus att eventuella fuktskador som inte omedelbart syns kan upptäckas. Samtidigt som undersökningsplikten ställer hårda krav på köparen leder den också ibland till orimliga resultat för säljaren. Även för den sistnämnde är det nämligen många gånger svårt att veta vilka brister hans fastighet är behäftad med.

9. Betänkande 1985/86:LU7
https://www.riksdagen.se/sv/dokument-och-lagar/dokument/betankande/om-vissa-fragor-betraffande-kop-av-smahus_g901lu7/

… Motionären påpekar vidare att undersökningsplikten samtidigt som den ställer hårda krav på köparen ofta leder till orimliga resultat för säljaren eftersom även den sistnämnda många gånger inte vet vilka brister hans fastighet har. Vidare väcker, enligt motionären, de senaste årens tragedier till följd av bl. a. **mögelhusskadorna** frågan vilket ansvar säljaren kan ha när hans hus är uppfört enligt gällande…

Motionären framhåller att de många fallen med s.k. **mögelhus** under senare är ofta orsakat svåra tolkningsproblem när det gäller innebörden av köparens undersökningsplikt. Enligt motionären är det för en vanlig husköpare ofta omöjligt att göra en sådan teknisk besiktning av ett hus att eventuella inte synliga fuktskador kan upptäckas.

10. Riksdagens protokoll 1986/87:35
https://www.riksdagen.se/sv/dokument-och-lagar/dokument/protokoll/riksdagens-protokoll-19868735_ga0935/

… Det har från bostadsdepartementets sida lagts ned mycken möda och mycket pengar när det gällt det skulle jag vilja säga ännu värre bekymret med **mögelhusen.** 76 Anf. 12 BIRGITTA HAMBRAEUS c. Herr talman. En av orsakerna till att man inte skriver så mycket om radonet är kanske att radon inte luktar. Man märker inte av det på samma sätt som man gör när det gäller mögel. Man kan förtränga radonproblemet. …

Jag kan säga till Birgitta Hambraeus att jag får mängder av brev från människor som skriver om sina bostadsbekymmer, sina **mögelhus** och hela denna problematik, men ytterligt få skriver om problem med radonhus och ekonomin….

Man kan naturligtvis diskutera om ett tilläggslån skall beviljas eller inte och hur stort ansvar som den enskilda människan skall ha resp. som samhället skall ha. Jag möter mycket ofta den frågan i samband med dyra ombyggnader av **mögelhus** - sådana ombyggnader kostar i genomsnitt 225 000 kr, men kan ibland gå upp till en halv miljon kronor. Det är aldrig så att den som köpt huset, byggt det eller svarat för det vill ta på sig denna kostnad, utan det är samhället som skall ha ansvaret härför. Men vi måste också själva bära ett visst ansvar för det som vi gör, och det måste också kommunerna göra.

11. Riksdagens protokoll 1987/88:40
https://www.riksdagen.se/sv/dokument-och-lagar/dokument/protokoll/riksdagens-protokoll-19878840-tisdagen-den-8_gb0940/

… I dag har såväl studenterna som 24 av de 60 anställda problem med irriterade ögon, huvudvärk och onormal trötthet, typiska **mögelhusproblem**. Inte bara högskolestyrelsen utan också bl. a. yrkesinspektionen, statshälsan och byggnadsstyrelsen anser att skolan bör flytta till nya lokaler. Trots de allvarliga hälso- och miljöproblemen och trots att nya lokaler inom kort finns tillgängliga har kraven på flyttning inte beaktats. …

Redan något år efter att högskolan i Halmstad 1982 flyttat in i sina nuvarande lokaler rapporterades att många bland personalen drabbats av hälsoproblem.

12. Betänkande 1987/88:BoU10
https://www.riksdagen.se/sv/dokument-och-lagar/dokument/betankande/om-anslag-till-bostadsforsorjningen-m.m.-prop_gb01bou10/

… Enligt uppgift motsvarar självrisken i genomsnitt endast en åttondel av genomsnittskostnaden för sanering av ett **mögelhus.** Efter bara två verksamhetsår är det emellertid ännu för tidigt att dra några säkra slutsatser av den minskade ärendetillströmningen. Enligt vad utskottet erfarit följer dock regeringen uppmärksamt utvecklingen på detta område. …

Utskottet finner det emellertid svårt att tro att en drabbad husägare skulle avstå från att åtgärda sitt hus och att söka stöd till följd av en självrisk motsvarande ett basbelopp.

13. Betänkande 1987/88:JuU21
https://www.riksdagen.se/sv/dokument-och-lagar/dokument/betankande/om-andringar-i-rattshjalpslagen-m.m.-prop_gb01juu21

… I avvaktan på att en ändrad ordning har åstadkommits bör enligt utskottets mening regeringen överväga om i **mögelhusmål** de rättssökande kan ges ett ökat ekonomiskt stöd i den i JuU 1987/88:21 46 riktning som har föreslagits i motion Jul5. Vad utskottet sålunda har uttalat bör riksdagen med anledning av motionerna Jul5 yrkandena 2 och 3Ju704 och Ju705 som sin mening ge regeringen till känna. dels att…

14. Riksdagens protokoll 1987/88:74
https://www.riksdagen.se/sv/dokument-och-lagar/dokument/protokoll/riksdagens-protokoll-19878874_gb0974/

…c till bostadsministern.om fukt- och mögelskadade bostadshus. *Orsaken till att riksdagen beslöt inrätta en fond för fukt- och mögelskador var att det är krångligt att reda ut ansvarsförhållandena, så att människor som drabbats av ett* ***mögelhus*** *snabbt kan få hjälp.* Antalet ansökningar hos småhusskadenämnden är lågt och minskade förra året, trots att många människor har stora svårigheter med kostnader för dubbel bosättning, därför att de inte kan bo i sitt **mögelhus** och inte har råd…

att reparera det

15. Riksdagens protokoll 1987/88:76
https://www.riksdagen.se/sv/dokument-och-lagar/dokument/protokoll/riksdagens-protokoll-19878876_gb0976/

… Det, är minimerat till ett basbelopp, en kostnad som enligt de normaluträkningar som gjordes före fastställandet motsvarar en åttondel av genomsnittskostnaden för saneringen av ett **mögelhus**. Jag tycker att vi har gått mycket långt. När vi givit oss in på att rätta till problemen i hus som är byggda för ett tjugotal år sedan har samhället tagit ett ansvar. I dessa tider när vi har så väldigt ont om pengar…

Birgitta Hambraeus, men vi kan inte lägga över allt ansvar på staten. Den enskilde som genomför affären måste också vara- beredd att ta ett visst ansvar i sammanhanget. Det är minimerat till ett basbelopp, en kostnad som enligt de normaluträkningar som gjordes före fastställandet motsvarar en åttondel av genomsnittskostnaden för saneringen av ett **mögelhus**.

16. Riksdagens protokoll 1987/88:99
https://www.riksdagen.se/sv/dokument-och-lagar/dokument/protokoll/riksdagens-protokoll-19878899-onsdagen-den-13_gb0999/

…I avvaktan på att något positivt kommer ut av detta menar vi reservanter att regeringen skall överväga ett ökat ekonomiskt stöd till rättssökanden i s. k. mögelhusmål. Med rättsskyddsförsäkringens maximibelopp om 75 000 kr. kommer man inte långt i komplicerade mögelhusmäl. Motion JU721 tar upp ett angeläget spörsmål om hur en felbehandlad patient kan hamna i svåra situationer på grund av att man nekas rättshjälp…

17. Riksdagens protokoll 1987/88:126
https://www.riksdagen.se/sv/dokument-och-lagar/dokument/protokoll/riksdagens-protokoll-198788126_gb09126/

…I avvaktan på att en ändrad ordning kommit till stånd ansåg riksdagen att regeringen borde överväga om de rättssökande ifall angående **mögelhus** kan ges ett ökat ekonomiskt stöd ur den statliga fonden för fukt- och mögelskador. Enligt vad jag erfarit har frågan angående fonden överlämnats till bostadsdepartementet. Det är angeläget att snabba besked ges till de personer som ligger i process om **mögelhus**…

…och har förbrukat rättsskyddet samt saknar andra medel att finansiera processerna. Utan stöd riskerar dessa personer att få lägga ner sina mål med därtill följande katastrofala konsekvenser för deras sociala och ekonomiska situation.

18. Riksdagens protokoll 1987/88:128
https://www.riksdagen.se/sv/dokument-och-lagar/dokument/protokoll/riksdagens-protokoll-198788128_gb09128/

… Riksdagen uttalade samtidigt att regeringen i avvaktan på ett sådant förslag bör överväga om de rättssökande i s. k. **mögelhusmål** kan ges stöd från den statliga fonden för fukt- och mögelskador. Fondstyrelsen kan enligt vad riksdagen tidigare har beslutat lämna stöd även till annat än reparationer av egnahem, om ändamålet med stödet är 75 Prot. 1987/88:128 förenligt med fondens syfte. …

Herr talman! Ingbritt Irhammar har frågat mig om när regeringen ämnar ge besked om på vilket sätt de som ligger i process om **mögelhus** skall kunna få nödvändigt stöd från staten till sina processkostnader. Bakgrunden till frågan är att riksdagen i april i år begärde att regeringen snarast lägger fram förslag som rör den allmänna rättshjälpen i miljömål.

19. Riksdagens protokoll 1988/89:12
https://www.riksdagen.se/sv/dokument-och-lagar/dokument/protokoll/riksdagens-protokoll-19888912_gc0912/

… Vi vill att våra barn skall bli en frisk generation. Minska allergierna genom att bygga hus som man kan leva i och vara frisk i. Inga **mögelhus**, radonhus, farliga träimpregneringsmedel. Inga flytspackelgolv, inga spånskivor som avger formaldehyd, inga heltäckningsmattor som innehåller en massa damm kvalster- som för resten det här huset verkligen är fullt med…

20. Riksdagens protokoll 1988/89:29
https://www.riksdagen.se/sv/dokument-och-lagar/dokument/protokoll/riksdagens-protokoll-19888929_gc0929/

… Vi är många som med spänd förväntan ser fram mot de initiativ som nu tydligen skall komma från regeringen. 38 AnL 36 Justitieminister LAILA FREIVALDS. Herr talman! Jag måste dess värre säga att jag inte känner igen den bild som målas upp här av situationen när det gäller **mögelhusen**. Det är riktigt att det finns en hel del mål som rör mögelskador i domstolarna för närvarande. Det är säkerligen också sant…

…att en hel del av dem är ganska gamla vid det här laget.

21. Riksdagens protokoll 1988/89:38
https://www.riksdagen.se/sv/dokument-och-lagar/dokument/protokoll/riksdagens-protokoll-19888938_gc0938/

… I praktiken är del i stället i stort sett mycket få människor som kan få hjälp. Till sist vill jag fråga bostadsministern Hur gör man, om man drabbas av ett **mögelhus** och det är konstaterat vilket i och för sig är ganska svårt, med 129 9 Riksdagens protokoll 1988189:36-38 Prot. 1988/89:38 5 december 1988 Om statliga kreditgarantier åt ägare till mögelskadade byggnader tanke på de luddiga bestämmelser…

Till sist vill jag fråga bostadsministern: Hur gör man, om man drabbas av ett **mögelhus** och det är konstaterat- vilket i och för sig är ganska svårt. Med tanke på de luddiga bestämmelser som finns och Socialstyrelsens rekommendationer; men trots allt inträffar ju sådant här ett antal gånger - att huset klassas som en sanitär olägenhet? Då måste ju huset evakueras. Man måste alltså flytta från sitt hus - dvs. man måste ha en ny bostad. Anser bostadsministern att det är självklart att myndigheterna i det

läget hjälper till med de dubbla hyrorna? Vem ställer egentligen upp'? Det skulle vara intressant att höra bostadsministerns svar.

22. Betänkande 1988/89:BoU2
https://www.riksdagen.se/sv/dokument-och-lagar/dokument/betankande/byggnaders-inomhusmiljo-m.m_gc01bou2/

… Självfallet skall alla onödiga avgivningar till inneluften från material, konstruktioner, inredning och verksamhet stoppas eller förhindras. Varför har det blivit så här Varför har vi så många fuktskador, **mögelhus**, luktande och missfärgade flytspackelhus Vems är felet Är det byggarnas, materialindustrins eller byggnormens fel Jag vill påstå att den främsta orsaken till de skador vi har i dag är okunnighet. Till en liten del är orsaken slarv och fusk. … (Ingemar Samuelsson)

Ingemar Samuelsson: Mina damer och herrar! Jag skall försöka ge teknikerns bild av problemets omfattning. Jag sysslar själv med skadeutredningar i hus som är drabbade av fukt och mögel. Statens provningsanstalt arbetar också med ventilationsproblem, undersökningar av radon, akustiska problem och problem i samband med kemisk avgivning från material. *Vad menar man med sjuka hus? Vanliga bekymmer i sådana hus är ohälsa och lukt.*

Ingjutna syllar är ett ur produktionssynpunkt lämpligt sätt att bygga snabbt. Men vi vet nu att även om syllarna är tryckimpregnerade så får man mögeltillväxt och lukt från dem.

Varför har det blivit så här? Varför har vi så många fuktskador, **mögelhus**, luktande och missfärgade flytspackelhus? Vems är felet! Är det byggarnas, materialindustrins eller byggnormens fel?

Tvärtom är *de flesta nybyggda hus både torrare och bättre än äldre hus, men de luktar.* Skälet kan vara - men vi är inte säkra på det ännu - att träet är mera benäget att lukta när det beväxes med mögel i nya konstruktioner än i äldre sådana.

Håkan Wahren: Mina damer och herrar! Jag arbetar på miljömedicinska enheten på Stockholms läns landsting och också för Socialstyrelsen. och jag har en bakgrund som hälsovårdsinspektör.

Den andra punkten gäller mögel, som finns i större eller mindre utsträckning i alla hus. Mögel skapar lukt och andra hälsoproblem. och det rör sig om minst 10 000 bostäder. Men vi vet inte säkert.

Vi har även den nyss avslutade utredningen om sunda och sjuka hus, vilken Arbetarskyddsstyrelsen, Socialstyrelsen och Planverket står bakom. I den utredningen finns det en rad förslag. Vi har den pågående allergiutredningen.

23. Betänkande 1988/89:JoU14
https://www.riksdagen.se/sv/dokument-och-lagar/dokument/betankande/livsmedelskontrollen_gc01jou14/

… Åtgärder m. m. för en bättre dricksvattenkvalitet mom. 11 Håkan Holmberg och Anders Castberger båda fp anför. Alla tecken tyder på att dricksvattensituationen i en nära framtid kan komma att bli akut och ett av de miljöproblem som vid sidan av **mögelhus** och radonfaran mest påverkar människors vardag och hälsa. Statistik från livsmedelsverket visar t. ex. att sedan 1979 mer än 25 000 människor i olika delar…

24. Proposition 1988/89:117
https://www.riksdagen.se/sv/dokument-och-lagar/dokument/proposition/om-rattshjalpens-organisation-m.-m_gc03117

… En sådan ändring bör i princip ta sikte på alla angelägenheter som omfattas av fastighetsundantaget, dvs. även mål med miljörättslig anknytning t.ex. enligt miljöskadelagen 1986:225 eller miljöskyddslagen 1969:387 och så kallade **mögelhusmål.** Vad frågan nu gäller är hur en regel som skall tillgodose detta önskemål närmare skall utformas. Utgångspunkten bör enligt min mening vara den principiella uppläggning av regeln som har föreslagits i promemorian. …

25. Motion 1988/89:Jo799 av Bengt Westerberg m.fl. (fp)
https://www.riksdagen.se/sv/dokument-och-lagar/dokument/motion/luften-och-havet_gc02jo799/

… Ett tankbilshaveri kan ju slå ut en vattentäkt totalt om det vill sig illa. Dricksvattensituationen kan bli akut i framtiden, det kan bli ett av de miljöproblem som vid sidan av **mögelhus** och radonfaran påverkar människors vardag mest. Statistik från livsmedelsverket visar t.ex. att sedan 1979 har mer än 25 000 människor infekterats av dåligt dricksvatten på olika håll i Sverige. I flera fall har det rört sig om magepidemier till följd av dåligt vatten. …

26. Motion 1988/89:So252 av Olof Johansson m.fl. (c)
https://www.riksdagen.se/sv/dokument-och-lagar/dokument/motion/handikappolitiken_gc02so252/

… Allergikernas tilltagande problem uppmärksammas bl.a. i en särskild utredning. Vi har krävt ökade insatser för sanering av s.k. mögelhus i en annan motion. Utbildning. Handikappade människor har särskilt stora behov av en kvalitativt och kvantitativt god utbildning, anpassad till deras behov. De kan annars drabbas av dubbla handikapp. Utbildningen bör leda till en yrkesutbildning som en grund för ett rikt vuxenliv. …

27. Riksdagens protokoll 1988/89:115
https://www.riksdagen.se/sv/dokument-och-lagar/dokument/protokoll/riksdagens-protokoll-198889115_gc09115/

… Ingen kan väl påstå att del är rent och snyggt i maktens boningar. Nej, de kan liknas vid ett **mögelhus.** Att påslå något annat skulle innebära att vi accepterar det som vi har sett komma fram i ljuset under de senaste åren. När del gäller konstitutionsutskottets möjligheter att granska vill jag säga att det inte bara handlar om utskottet. …

Sverige må se lugnt och välmående ut på ytan. I själva verket håller delar av vårt rättssystem och vår maktutövning på att ruttna inifrån. Sverige är som ett ***mögelhus****. Det luktar inte bra.*

Jag är, herr talman, inte heller samma panikmakare som Anders Björck. Jag tror inte att Sverige håller på att bli ett **mögelhus**. Jag tror inte heller att nomenklaturen håller på att ta över.

Till Bo Hammar vill jag gärna säga att visst kan man likna maktens boningar vid ett **mögelhus**.

För någon halvtimme sedan utnämnde Anders Björck det svenska rättssamhället till ett **mögelhus**. Jag vill inte förneka att Ebbe Carlsson-affären är ett tecken på att det sprider sig mögel litet här och var på samhällets toppnivå.

28. Riksdagens protokoll 1988/89:119
https://www.riksdagen.se/sv/dokument-och-lagar/dokument/protokoll/riksdagens-protokoll-198889119_gc09119/

… Under en tid byggdes del jordvärme. Det isolerades hus med treglasfönster, vilket ledde till freonutsläpp och **mögelhus.** För några är sedan var det ingen konst att avveckla kärnkraften, eftersom de förnybara energikällorna hade kommit längre sade man i sin tekniska och kommersiella utveckling än vad man tidigare sade sig ha trott Sedan fattades beslut om att förtidsavveckla kärnkraft 1995 och 1996, oavsett hur den skulle ersättas. …

29. Riksdagens protokoll 1988/89:129
https://www.riksdagen.se/sv/dokument-och-lagar/dokument/protokoll/riksdagens-protokoll-198889129_gc09129/

… Men varför det. Det finns väl andra lösningar ute i världen. Det är inte alls säkert att andra länder har så många **mögelhus** som Sverige har jag talar då om hus med samma standard i övrigt. Per Gahrton kan inte få in i huvudet att det finns andra bra lösningar och att vi kan ta del av dem under en anpassningsprocess. Jag tänker på en annan reservation, där miljöpartiet talar om utvecklingen i fråga om att…

30. Betänkande 1989/90:BoU3
https:/www.riksdagen.se/sv/dokument-och-lagar/dokument/betankande/egenskapskrav-pa-byggnader-m.m_gd01bou3

… Även om stöd kan utgå från småhusskadenämnden och fonden är detta stöd enligt motionären inte alltid tillgängligt eller tillräckligt. För att förbättra situationen för dem som drabbats av **mögelhus** förordas därför att statliga kreditgarantier för banklån skall införas i dessa fall. Även i motion 1988/89:Bo545 fp tas frågan om ett utökat stöd till dem som drabbats av **mögelhus** upp. Enligt motionären…

31. Motion 1989/90:Ju301 av Ingbritt Irhammar och Marianne Andersson
https://www.riksdagen.se/sv/dokument-och-lagar/dokument/motion/rattsvasendets-kompetens-betraffande-miljobrott_gd02ju301/

…Bl.a. skall allmän rättshjälp kunna beviljas i sådana fastighetstvister, där den rättssökandes kostnader kan uppgå till betydande belopp, t.ex. i miljömål och i mål om **mögelhus**, och där rättsskyddsförsäkringen inte räcker för att betala den rättssökandes egna kostnader. Det finns dock fortfarande anledning att förbättra rättshjälpen ytterligare och genomföra miljöskadeutredningens förslag om att införa…

32. Motion 1989/90:So299 av Ingbritt Irhammar och Marianne Andersson
https://www.riksdagen.se/sv/dokument-och-lagar/dokument/motion/ekonomiskt-bistand-till-mogeldrabbade-familjer_gd02so299/

…Sålunda bör familjer, som på grund av fukt och mögel i sina hus, tvingas flytta ur och hyra bostad på annat håll under t.ex. saneringsarbete i **mögelhuset**, kunna betraktas som katastrofärende vad gäller socialtjänstlagen. Det är helt orimligt, att dessa familjer skall tvingas stå för ränta och amortering på det mögeldrabbade huset samtidigt som de får betala hyran i sin nya bostad. …

33. Motion 1990/91:Jo792 av Ingbritt Irhammar och Karin Starrin (c)
https://www.riksdagen.se/sv/dokument-och-lagar/dokument/motion/miljolagstiftningen_ge02jo792/

…Bl.a. skall allmän rättshjälp kunna beviljas i sådana fastighetstvister, där den rättssökandes kostnader kan uppgå till betydande belopp, t.ex. i miljömål och i mål om **mögelhus**, och där rättsskyddsförsäkringen inte räcker för att betala den rättssökandes egna kostnader. Det finns dock fortfarande anledning att förbättra rättshjälpen ytterligare och genomföra miljöskadeutredningens förslag om att införa…

34. Motion 1990/91:Ub208 av Eva Goës m.fl. (mp)
https://www.riksdagen.se/sv/dokument-och-lagar/dokument/motion/elevernas-arbetsmiljo_ge02ub208/

…Vem för barnens talan. Vart tredje barn drabbas av allergi, vilket kan bero på en kombination av faktorer i yttre och inre miljö t ex avgaser från trafik och **mögelhusmiljö** och skolornas miljö är i allra högsta grad bidragande orsak till ökningen av allergier. Förra årets riksdagsbeslut om att elevsamverkan i arbetsmiljöarbetet skall utökas är bra. Inte bara gymnasiet och högstadiet utan hela grundskolan omfattas av miljöskyddet…

35. Motion 1990/91:Jo113 av Olof Johansson m.fl. (c)
https://www.riksdagen.se/sv/dokument-och-lagar/dokument/motion/med-anledning-av-prop.-19909190-en-god-livsmiljo_ge02jo113

…Bl.a. skall allmän rättshjälp kunna beviljas i sådana fastighetstvister, där den rättssökandes kostnader kan uppgå till betydande belopp, t ex i miljömål och i mål om **mögelhus**, och där rättsskyddsförsäkringen inte räcker för att betala den rättssökandes egna kostnader. Det finns dock fortfarande anledning att förbättra rättshjälpen ytterligare och genomföra miljöskadeutredningens förslag om att införa…

36. Betänkande 1990/91:JuU27
https://www.riksdagen.se/sv/dokument-och-lagar/dokument/betankande/anslag-till-rattshjalp-m.m_ge01juu27

…En sådan ändring borde enligt propositionen s. 22 i princip ta sikte på alla angelägenheter som omfattas av fastighetsundantaget, dvs. även mål med miljörättslig anknytning, t.ex. enligt miljöskadelagen eller miljöskyddslagen, och s.k. **mögelhusmål**. Med hänsyn till redan existerande möjligheter att erhålla rättshjälp samt till de begränsade resurser som finns tillgängliga saknas enligt utskottet förutsättningar…

37. Motion 1990/91:So88 av Gunnar Björk m.fl. (c, m, fp, v, s, mp)
https://www.riksdagen.se/sv/dokument-och-lagar/dokument/motion/med-anledning-av-prop.-199091175-vissa_ge02so88

… Sedan mitten av 1970- talet är statens institut för byggnadsforskning, SIB, lokaliserat till Gävle. Ett viktigt arbetsområde för SIB är bl.a. att komma till rätta med problemen med **fukt- och mögelhus** och att i övrigt bostadsbyggandet sker i sådana former att folkhälsan gagnas. Här föreligger ett synnerligen stort och betydelsefullt samarbetsområde mellan folkhälsoinstitutet och SIB. Folkhälsoinstitutet…

kan komma att verka såväl som beställare av forskningsuppgifter hos SIB som förmedlare av de forskningsresultat som SIB lägger fram. Det nära samarbetet skulle ofrånkomligen gynnas av en lokalisering till samma ort.

38. Motion 1991/92:So523 av Karin Starrin m.fl. (c, m, fp, v, s, kds, nyd)
https://www.riksdagen.se/sv/dokument-och-lagar/dokument/motion/folkhalsoinstitutet-till-gavleborg_gf02so523/

… Sedan mitten av 1970- talet är statens institut för byggnadsforskning, SIB, lokaliserat till Gävle. Ett viktigt arbetsområde för SIB är bl.a. att komma till rätta med problemen med **fukt- och mögelhus** och att i övrigt bostadsbyggandet sker i sådana former att folkhälsan gagnas. Här föreligger ett synnerligen stort och betydelsefullt samarbetsområde mellan folkhälsoinstitutet och SIB. Folkhälsoinstitutet…

39. Motion 1993/94:Bo234 av Eva Zetterberg och Lars Werner (v)
https://www.riksdagen.se/sv/dokument-och-lagar/dokument/motion/kvalitetsmarkning-av-bostader_gh02bo234/

… En bostad däremot kan ge de boende cancer utan att för den skull behöva besiktigas eller ännu mindre åtgärdas. Problemen med sjuka hus är välkända radonhus, flytspackel, **mögelhus,** PVS-material, bristande ventilation, etc. De boende eller bostadssökande har ofta svårt att bedöma en lägenhets kvalitet om man inte har specialkunskaper. Bostäder borde kvalitetsmärkas med ett system som är begripligt och tillåter jämförelser mellan olika bostäder. …

40. Motion 1993/94:Jo645 av Siw Persson (fp)
https://www.riksdagen.se/sv/dokument-och-lagar/dokument/motion/miljorisker-vid-pappersframstallning_gh02jo645/

…Det återvunna papperet kan bära okända mikro-organismer och lösningsmedelsfria färger bubbla av mögel. Vi kan stå inför ett nytt **mögelhusproblem**. Idag är återvinning av papper och utveckling av nya lösningsmedelsfria färger steg in i ett miljövänligare samhälle. Tekniker, ytkemister och återvinningsexperter i olika led presenterar nya lösningar. Tekniskt och ekonomiskt är de nya processerna bra, men man har glömt ett led, nämligen att konsultera mikrobiologerna…

41. Motion 1994/95:Bo514 av Owe Hellberg m.fl. (v)
https://www.riksdagen.se/sv/dokument-och-lagar/dokument/motion/kvalitetsmarkning-av-bostader_gi02bo514/

… Motion till riksdagen 1994/95:Bo514 av Owe Hellberg m.fl. v Kvalitetsmärkning av bostäder. Krav på miljömärkning av olika produkter har blivit en allt viktigare fråga för konsumenterna, den borde gälla även bostäder. Problemen med sjuka hus är välkända radonhus, flytspackel, **mögelhus,** PVS-material, bristande ventilation m.m. De boende har ofta svårt att bedöma en lägenhets kvalitet om man inte har specialkunskaper…

42. Riksdagens protokoll 1996/97:42
https://www.riksdagen.se/sv/dokument-och-lagar/dokument/protokoll/riksdagens-snabbprotokoll-19969742-tisdagen-den_gk0942/

… Omläggningen av stödvillkoren bör finansieras inom den ekonomiska ramen för stödet från fonden. Dessutom måste man satsa på mer information om stödmöjligheten. Människor i s.k. **mögelhus** skall inte behöva försöka dölja sina problem i stället skall samhället göra det möjligt för de utsatta att snabbt kunna åtgärda skadorna och komma till rätta med orsakerna. 10. Till sist, herr talman, vill jag nämna bostads- bidragen och förändringen av dessa. …

43. Riksdagens protokoll 1999/2000:38
https://www.riksdagen.se/sv/dokument-och-lagar/dokument/protokoll/riksdagens-snabbprotokoll-1999200038-torsdagen_gn0938/

…Det är att tala om radonfaran, om slum och gräddhyllor… Det är att tala om utförsäljning av kommunala bostäder till spekulativa förvärvare eller tvångströjor på fastighetsägare som vill förändra sitt bostadsbestånd. Det är att tala om **fukt- och mögelhus.** Det är att tala om radonfaran, om slum och gräddhyllor. Det är att tala om stora skillnader. Det är att tala om olika förutsättningar, om orättvisor, om trygghet för vissa och otrygghet för andra, om en…

44. Statens offentliga utredningar 2001:95
https://www.riksdagen.se/sv/dokument-och-lagar/dokument/statens-offentliga-utredningar/sou-2001-95-d5_gpb395d5/

…Han tjänar pengar på att hyra ut **mögelhus** till folk som inte har några bostäder. Det var ju pension för min del va. Och jag sköter hela den biten själv va, med hyresinbetalningarna. Han tjänar pengar på att hyra ut **mögelhus** till folk som inte har några bostäder. CL Hur länge bodde du på det stället IP11: Där bodde jag nästan två år. Ett och ett halvt drygt. CL Och det fungerade bra tills det att han skulle renovera IP11: Utmärkt. Javisst. …

45. Riksdagens protokoll 2001/02:93
https://www.riksdagen.se/sv/dokument-och-lagar/dokument/protokoll/riksdagens-snabbprotokoll-20010293-onsdagen-den_gp0993/

… De tycker tydligen att det inte är några fel på de regler som vi har. Ändå bygger vi inte fullt ändamålsenliga bostäder. Vi får väl fortsätta att fundera på det här. I **mögelhusens** spår, för att fortsätta att vara historisk, tog vi i Center-partiet också upp kravet på att införa en kvalitetsgaranti för bostäder för att därmed göra miljön till en konkurrensfaktor på marknaden. Jag konstaterar att det kravet fortfarande äger giltighet. …

46. Betänkande 2001/02:FIU21
https://www.riksdagen.se/sv/dokument-och-lagar/dokument/betankande/tillaggsbudget-1-for-budgetaret-2002-prop_gp01fiu21/

… Det borde ni borgerliga ta upp en och annan gång. *Nyligen dokumenterades ett antal upprörande fall där fusk och slarv har varit legio -* ***mögelhus,*** *fallfärdiga broar, asfaltskarteller osv.* Nu genomför vi en rad åtgärder som på intet sätt kommer att lösa alla problemen men som sannolikt kommer att bidra till förbättringar. Investeringsstöd för byggande ger i praktiken samma effekter som en lägre moms. …

47. Riksdagens protokoll 2001/02:123
https://www.riksdagen.se/sv/dokument-och-lagar/dokument/protokoll/riksdagens-snabbprotokoll-200102123-onsdagen-den_gp09123/

…Det borde ni borgerliga ta upp en och annan gång. *Nyligen dokumenterades ett antal upprörande fall där fusk och slarv har varit legio - mögelhus, fallfärdiga broar, asfaltskarteller osv.* Nu genomför vi en rad åtgärder som på intet sätt kommer att lösa alla problemen men som sannolikt kommer att bidra till förbättringar. Investeringsstöd för byggande ger i praktiken samma effekter som en lägre moms. …

48. **Statens offentliga utredningar 2004:47**
https://www.riksdagen.se/sv/dokument-och-lagar/dokument/statens-offentliga-utredningar/naringslivet-och-fortroendet-del-3_gsb347d3/

…Om detta ändå, mot all förmodan, inte skulle vara fallet, *står vi inför det som vi brukar kalla en skandal, läkarna som styckar kroppen, präster som våldtar småpojkar, bankdirektörer som fifflar,* ***mögelhus*** *m.m.* Dialog och förtroende Den tyske filosofen Jürgen Habermas betonar förtroende som en förutsättning för samtal. Vi förutsätter ju normalt enkel tillit att den andre menar det hon säger sanning och att…

49. Interpellation 2007/08:342 av Moberg, Carina (s)
https://www.riksdagen.se/sv/dokument-och-lagar/dokument/interpellation/regeringens-bostadspolitiska-rivningspolitik_gv10342/

…Det är inte alls ett sätt att släta över något annat, utan det är bara att konstatera att ska vi nu gå in i en fas när vi ska bygga energieffektiva hus så behövs kunskap. Det finns en inneboende rädsla att man inte får misslyckas och göra fel och få tillbaka något slags **mögelhustänkande** och sådant. Det finns system av typen passivhus som kräver en väldigt exakt teknik, och den tekniken måste branschen naturligtvis….

50. Riksdagens protokoll 2007/08:62
https://www.riksdagen.se/sv/dokument-och-lagar/dokument/protokoll/riksdagens-protokoll-20070862-tisdagen-den-12_gv0962/

…Det är inte alls ett sätt att släta över något annat, utan det är bara att konstatera att ska vi nu gå in i en fas när vi ska bygga energieffektiva hus så behövs kunskap. Det finns en inneboende rädsla att man inte får misslyckas och göra fel och få tillbaka något slags **mögelhustänkande** och sådant. Det finns system av typen passivhus som kräver en väldigt exakt teknik, och den tekniken måste branschen naturligtvis…
